# FTO Knockout Causes Chromosome Instability and G2/M Arrest in Mouse GC-1 Cells

**DOI:** 10.3389/fgene.2018.00732

**Published:** 2019-01-21

**Authors:** Tao Huang, Qiang Gao, Tongying Feng, Yi Zheng, Jiayin Guo, Wenxian Zeng

**Affiliations:** Laboratory of Reproductive Biology and Cell Engineering, College of Animal Science and Technology, Northwest A&F University, Xianyang, China

**Keywords:** *N*^6^-methyladenosine, FTO, spermatogonia, cell cycle, chromosome instability, mitotic checkpoint

## Abstract

*N*^6^-methyladenosine (m^6^A) is the most abundant modification on eukaryotic mRNA. m^6^A plays important roles in the regulation of post-transcriptional RNA splicing, translation, and degradation. Increasing studies have uncovered the significance of m^6^A in various biological processes such as stem cell fate determination, carcinogenesis, adipogenesis, stress response, etc, which put forwards a novel conception called epitranscriptome. However, functions of the fat mass and obesity-associated protein (FTO), the first characterized m^6^A demethylase, in spermatogenesis remains obscure. Here we reported that depletion of FTO by CRISPR/Cas9 induces chromosome instability and G2/M arrest in mouse spermatogonia, which was partially rescued by expression of wild type FTO but not demethylase inactivated FTO. FTO depletion significantly decreased the expression of mitotic checkpoint complex and G2/M regulators. We further demonstrated that the m^6^A modification on Mad1, Mad2, Bub1b, Cdk1, and Ccnb2 were directly targeted by FTO. Therefore, FTO regulates cell cycle and mitosis checkpoint in spermatogonia because of its m^6^A demethylase activity. The findings give novel insights into the role of RNA methylation in spermatogenesis.

## Introduction

Life-long male fertility relies on spermatogenesis that is responsible for the generation of millions of sperm (Fok et al., 2014). Spermatogenesis is a complex developmental process that consists of three stages: mitosis of spermatogonia, meiosis of spermatocyte and transformation of sperm from haploid spermatids (Kanatsu-Shinohara and Shinohara, 2013). Thus, spermatogonia are the cornerstone of sperm production (Hamra et al., 2004). Nevertheless, the underlying mechanism regulating spermatogonial proliferation and differentiation remains largely elusive.

Over 100 different types of chemical modifications have been found in RNAs, among which the *N*^6^-methyladenosine (m^6^A) is mostly prevalent in eukaryotes (Niu et al., 2013). In general, m^6^A is mainly enriched near the stop codon, within the consensus motif DRACH (D = A, G, U; R = A, G; H = A, C, U) (Dominissini et al., 2012). In most species, m^6^A is installed by the “writer” complex that is composed of METTL3, METTL14, WTAP, and several unknown components (Liu et al., 2014). Interestingly, m^6^A can be erased by the demethylase FTO and ALKBH5 (Zheng et al., 2013), and is recognized by the YTH-domain-containing proteins (Zhu et al., 2014). Growing evidences have indicated that m^6^A is involved in post-transcriptional processes including translation, mRNA degradation, alternative splicing, and microRNA maturation, thus affecting gene expression (Wang et al., 2014, 2015; Alarcon et al., 2015). Recent studies have elucidated the significance of m^6^A in the regulation of stem cell fate determination, pre-adipocytes differentiation, DNA damage response, self-renewal of neural stem cells, and T cell homeostasis (Batista et al., 2014; Zhao et al., 2014; Li H.B. et al., 2017; Xiang et al., 2017; Wang et al., 2018).

The importance of m^6^A in spermatogenesis have been preliminarily revealed. Depletion of METTL3 leads to inhibition of spermatogonial differentiation and arrest of meiosis initiation, resulting in infertility (Xu et al., 2017). Furthermore, double knockout of METTL3 and METTL14 in advanced germ cells with Stra8-Cre disrupts spermiogenesis (Lin et al., 2017). Knockout of ALKBH5 causes severe apoptosis in spermatogonia and spermatocytes (Zheng et al., 2013). Consistently, YTHDC2 deficient leads to arrest of meiosis at zygotene spermatocytes (Hsu et al., 2017). These evidences provide the proof of concept with respect to the involvement of m^6^A in spermatogenesis.

The fat mass and obesity-associated (*Fto*) gene, located at chromosome 16 in humans, encodes FTO protein, which belongs to the α-ketoglutarate-dependent dioxygenase alkB family. Loss of FTO leads to postnatal growth retardation and a significant reduction in adipose tissue and lean body mass (Fischer et al., 2009). It has been sufficiently demonstrated that FTO possesses the activity of m^6^A demethylase, which regulates pre-adipocyte differentiation, the leukemic oncogene-mediated cell transformation, and tumorigenesis of glioblastoma stem cells (Li L. et al., 2017; Li Z.J. et al., 2017). Recent studies have revealed the significance of FTO in the regulation of neural development and stress response (Du et al., 2018; Engel et al., 2018). Interestingly, two missense mutations in *Fto* are associated with the reduced semen quality in azoospermic patients (Landfors et al., 2016). However, little is known about the functions of FTO in spermatogenesis.

The aim of the present study was to gain more insights into the role of FTO in spermatogonia division. To this end, we employed the mouse GC-1 spermatogonial cell line as a research model and performed loss-of-function study by CRISPR/Cas9. We found that knockout of *FTO* triggered abnormal chromosome segregation and cell cycle arrest. This phenotype could be partially rescued by wild-type FTO but not mutant FTO. FTO depletion elevated the m^6^A level of core mitosis checkpoint complex (MCC) components and G2/M regulators. Therefore, FTO regulates cell cycle and mitosis checkpoint in spermatogonia because of its m^6^A demethylase activity.

## Materials and Methods

### Cell Culture and Plasmid Transfection

The mouse spermatogonia cell line (GC-1) were maintained in Dulbecco’s Modified Eagle’s Medium (GE) with 10% fetal bovine serum (Gibco), 100 U/ml penicillin and 0.1 mg/ml streptomycin (PS) and incubated at 37°C with 5% CO_2_. For plasmid transfection, cells were seeded to 6-well plate (2 × 10^5^ cells per plate) and cultured overnight. Plasmids were transfected to cells using TurboFect^TM^ Transfection Reagent (Thermo Fisher Scientific^TM^) following the instructions. Twenty-four hours post-transfection, cells were subjected to puromycin (2 μg/ml, Sigma) selection for 2 days.

### Antibodies

The primary and secondary antibodies were purchased from commercial sources as follows: Mouse anti-FTO, Mouse anti-Mad2, Mouse anti-Cdc20, Mouse anti-Bub1, Mouse anti-Bub1b, Mouse anti-Bub3, Mouse anti Tubulin (Santa Cruz Biotechnology), Rabbit anti m^6^A (Synaptic Systems), Rabbit anti-Actin (Sigma-Aldrich). HRP-goat anti rabbit IgG (CWbio) and HRP-goat anti mouse IgG (CWbio).

### Vectors Construction

For knocking out FTO in GC-1 cells, the following sgRNAs were designed and synthesized, sg-FTO1U: 5′-ACCGCCGTCCTGCGATGATGAAG-3′, sg-FTO1D: 5′-AAACCTTCATCATCGCAGGACGG-3′, sg-FTO2U: 5′-ACCGGAACTCTGCCATGCACAG-3′, sg-FTO2D: 5′-AAACCTGTGCATGGCAGAGTTC-3′. The PGL3-U6-PGK plasmid (gifted from Shanghai Tech University) was used as the backbone. Plasmid was ligated with annealed sgRNAs via T4 ligase (Thermo Fisher Scientific). For the FTO rescue experiment, total RNA was extracted from GC-1 cells using RNAiso plus Reagent (Takara Clontech). cDNA was synthesized by the first strand cDNA synthesis kit (Takara Clontech) following the manufacturer’s instructions. The following primers were designed and synthesized for the amplification of FTO CDS, FTO-res-F: 5′-GAATCTAGAATGAAGCGCGTCCAGAC-3′, FTO-res-R: 5′-GGAGAATTCTGCTGGAAGCAAGATCCTAG-3′. PCR products were purified by the PCR clean-up Kit (Axgen). CD513B plasmid and purified PCR products were digested by restriction enzymes *Eco*RI and XbaI (NEB), following by ligation using the T4 ligase.

For the FTO mutant experiment, the following primers were designed and synthesized. FTO-mut-1F: 5′-GAATCTAGAATGAAGCGCGTCCAGAC-3′, FTO-mut-1R: 5′-GCGTGAGTGGAACTAAACGCAGGCTGTGAGCCAGC-3′, FTO-mut-2F: 5′-GCTGGCTCACAGCCTGCGTTTAGTTCCACTCACCG-3′, FTO-mut-2R: 5′-GGAGAATTCTGCTGGAAGCAAGATCCTAG-3′. cDNA of FTO was used as the PCR template. PCR products were purified using Gel Extraction Kit (Omega) following by recombinant using Neotec reagent. Recombined fragments were purified and ligated with CD513-B1 plasmids.

### T7E1 Assay

Genomic DNA was extracted using phenol-chloroform followed by ethyl alcohol precipitation. For indels detection, following primers were designed and synthesized, FTO-F: 5′-CCAGTGTCTCGCATCCTCATC-3′, FTO-R: 5′-TTACTCATCCTCAGAGCCTCAGA-3′. PCR products were purified using PCR clean up Kit. The purified DNA was annealed following by digested by T7 endonuclease (NEB). After digestion at 37°C for 30 min, DNA was analyzed by the agarose gel electrophoresis. Image J was used to calculate the cleavage efficiency.

### Establishment of the FTO^−/−^ Cell Strain

Plasmids expressing cas9 and sgRNAs were co-transfected to spermatogonia using the TurboFect^TM^ Transfection Reagent as previously described. Twenty-four hours post-transfection, cells were screened using 2 μg/ml puromycin for 2 days. The residual cells were suspended to 300 cells/ml and seeded to the 100-mm-dish. After 7 days of culture, mono clones were observed under the microscope. Monoclones were picked and transferred to the 96-well plate (one clone per well) followed by a 7-day culture. Subsequently, genomic DNA of each cell clone were extracted using the QuickExtract^TM^ DNA Extraction Solution 1.0 (Epicenter) following the manufacturer’s instructions. The DNA fragments containing sgRNA target sites were amplified using PCR followed by Sanger sequencing. Cell strains harboring frameshift mutations within *Fto* locus in di-alleles were considered as the Fto^−/−^ cell strain.

### m^6^A Dot Blot

Total RNA was extracted from cells using Trizol reagent (TAKARA). mRNA was isolated and purified using Poly Attract mRNA Isolation System III with Magnetic Stand (Promega) following the manufacturer’s instructions. For m^6^A dot blot, mRNA was hybridized onto the Hybond-N+ membrane (GE Healthcare). After crosslinking at 80°C for 30 min, the membrane was blocked with 5% non-fat milk (Bio-Rad) for 1 h, incubated with rabbit anti-m^6^A antibody (1:1000, Synaptic Systems) at 4°C overnight. Then the membrane was incubated with HRP-conjugated goat anti-rabbit IgG at room temperature for 2 h. After being incubated with Immobilon Western Chemiluminescent HRP Substrate (Millipore), the immunocomplex was photographed using the ECL imaging system (Bio-Rad). Finally, the membrane was stained with 0.02% methylene blue to eliminate the difference in mRNA amount. Relative m^6^A level was quantified via gray intensity analysis using ImageJ.

### Western Blot Assay

Cells were lysed with RIPA buffer containing 1% PMSF followed by ultrasonication. Cell lysates were incubated on ice for 30 min, centrifuged at 10,000 *g* for 10 min. The supernatants were collected and the protein concentration was detected using a BCA detection Kit. Equal amount of proteins was loaded to the polyacrylamide gel. The proteins were separated through SDS-PAGE using the electrophoresis apparatus (Bio-Rad). After electrophoresis, the proteins were transferred to the PVDF membrane (Millipore, IBFP0785C) using a semi-dry transfer instrument (Bio-Rad). The membranes were blocked with 5% non-fat milk for 1 h at room temperature, incubated with primary antibodies at 4°C overnight. Subsequently, the membranes were washed with PBST and incubated with HRP-conjugated secondary antibodies for 1 h at room temperature. After washing, the membranes were incubated with the Immobilon Western Chemiluminescent HRP Substrate (Millipore, United States) and photographed using the ECL imaging system (Bio-Rad, United States).

### Flow Cytometric Analysis

For cell cycle analysis, cells were suspended in 75% cold ethanol and treated with 0.1% Triton X-100 and 100 μg /ml RNase at 37°C for 30 min. Subsequently, the cells were stained with 50 μg/ml PI for 2 h and analyzed by flow cytometry. For cell clustering analysis, cells were fixed in cold 70% ethanol, permeablized with 0.1% Triton X-100. Then the cells were stained with 4′,6-diamidino-2-phenylindole (DAPI, Thermo Fisher Scientific) for 30 min and analyzed by flow cytometry.

### Quantitative Real-Time PCR

Cells were lysed with Trizol regent (TAKARA). Total RNA was isolated by chloroform followed by precipitating with isopropanol. cDNA was synthesized with the PrimeScript^TM^ RT reagent Kit (TAKARA) following the manufactory’s instructions. Primers designed and synthesized for RT-qPCR were listed in Supplementary Table S1. Quantitative PCR was performed using the SYBR Green II PCR Mix (TAKARA) and the IQ5 (Bio-Rad).

### Chromosome Spread Assays

Wild-type and FTO-KO cells were cultured in complete medium to 70% confluence and treated with 50 ng/μL nocodazole for 16 h. Cells were collected and subjected to hypotonical swell in 75 mM KCl at 37°C for 30 min. Subsequently, cells were fixed in Carnoy’s fluid (methanol: acetic acid 3:1) at room temperature for 30 min. Cells were dropped onto pre-cooling glass slides and air dried. Slides were stained with Hochest 33342 (1:500) and photographed under the fluorescence microscope. For each biological repetition, chromosome number of 150 cells were counted and analyzed.

### Immunofluorescence

For immunofluorescence analysis, cells were fixed in 4% paraformaldehyde/PBS for 30 min, permeabilized in 0.5% Triton X-100/PBS and blocked with 5% bovine serum albumin (BSA). After washed with PBS for three times, the cells were incubated with rabbit anti-CREST antibody (1:200) and mouse anti β-tubulin (1:200) antibody at 4°C overnight. Then the cells were washed for another three times with PBS and incubated with FITC-conjugated goat anti rabbit and rhodamine red-conjugated goat anti-mouse secondary antibodies (1:2000) at room temperature for 1 h. Cells were washed in PBS for three times and counterstaining with DAPI. Images were photographed under an inverted fluorescence microscope (Olympus, IX71).

### m^6^A-IP-qPCR

Total RNA was extracted from cells using the RNAiso plus regent (TAKARA). mRNA was isolated using the PolyATtract^®^mRNA Isolation Systems (Promega, Z5310) following the manufacturer’s instructions. The m^6^A-IP was performed as previously described (Dominissini et al., 2012). In brief, 3 μg mRNA was mixed with 12.5 μL of rabbit anti m^6^A antibody (0.5 mg/mL, Synaptic Systems, 202003), 5× IP buffer (50 mM Tris–HCl, pH 7.4, 750 mM NaCl, and 0.5% NP-40), RNA inhibitor and DEPC-treated nuclease free water to make 500 μL of IP mixture. Protein A beads were washed with wash buffer (IP buffer mixed with RNA inhibitor) for three times, and then blocked with 0.5 mg/mL BSA. After blocking, the beads were incubated with IP mixture and rotated at 4°C overnight followed by extensive washing. Bound RNA was eluted using 100 μL elution buffer (1× IP buffer, 6.7 mM m^6^A). For m^6^A level measurement, 40 ng of IP-RNA and Input-RNA was used for cDNA synthesis. The m^6^A^+^ mRNA level was finally determined by real-time quantitative PCR.

### RNA-Decay Assay

WT cells and FTO-KO cells were treated with 5 μg/mL actinomycin D for 0, 3, and 6 h, respectively. Cells were harvested and subjected to RNA extraction. Real-time quantitative PCR were used to analyze the mRNA level of target genes in each group.

### Statistical Analysis

All data were collected from at least three independent experiments. Data were analyzed using two-tailed Student’s *t*-test or one-way ANOVA followed by a Duncan’s multiple range test (SPSS 22 for windows). Significance were presented as ^∗^*p* < 0.05, ^∗∗^*p* < 0.01, and ^∗∗∗^*p* < 0.001. Error bars represented SEM of the mean.

## Results

### Depletion of FTO in Spermatogonia Subjected to CRISPR-Cas9 Gene Editing

**FIGURE 1:**
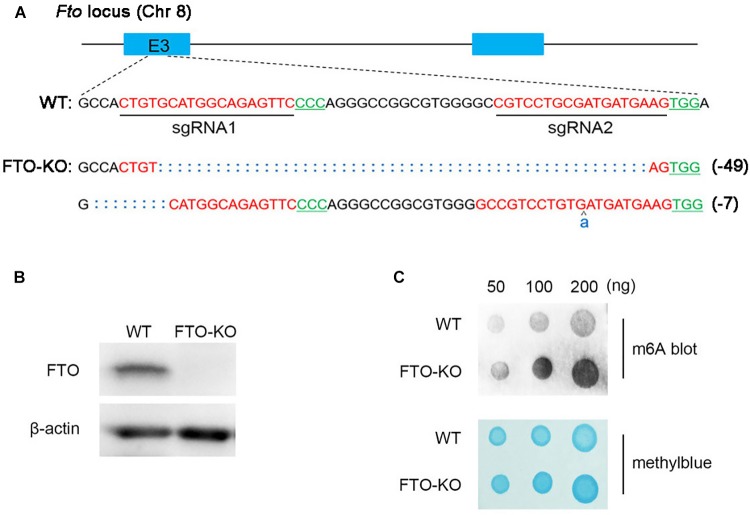
Depletion of fat mass and obesity-associated protein (FTO) in spermatogonia via CRISPR/Cas9 system. **(A)** Schematic diagram of sgRNAs designed for FTO knockout. Sequences of modified *Fto* loci in FTO-KO cells. **(B)** Western blot analysis of FTO expression in wild-type cells and FTO-KO cells. β-actin was used as the loading control. **(C)** m^6^A dot blot analysis of wild-type cells and FTO-KO cells. Methyl blue stain was used to eliminate the difference in RNA amount.

The endogenous FTO of spermatogonia was abrogated using the CRISPR-Cas9 genome editing technique with *Fto*-specific sgRNAs. Two sgRNAs targeting exon 3 of *Fto* were designed and synthesized (Figure 1A). The CRISPR-Cas9 mutations resulted in 7 and 49 nucleotide deletions in the two alleles of the *Fto* gene, respectively, and thus induced the frameshift mutations at the target sites (Figure 1A). Western blot analysis showed that the FTO protein was completely absent in the KO cells (Figure 1B). We next detected total m^6^A level of mRNA extracted from wild type cells and FTO-KO cells. The blot signal strength showed significant increases in mRNA of FTO depletion cells (Figure 1C), indicating that depletion of FTO elevated the m^6^A level in spermatogonia.

### FTO Depletion Induces Formation of Multinuclear Giant Cells

**FIGURE 2:**
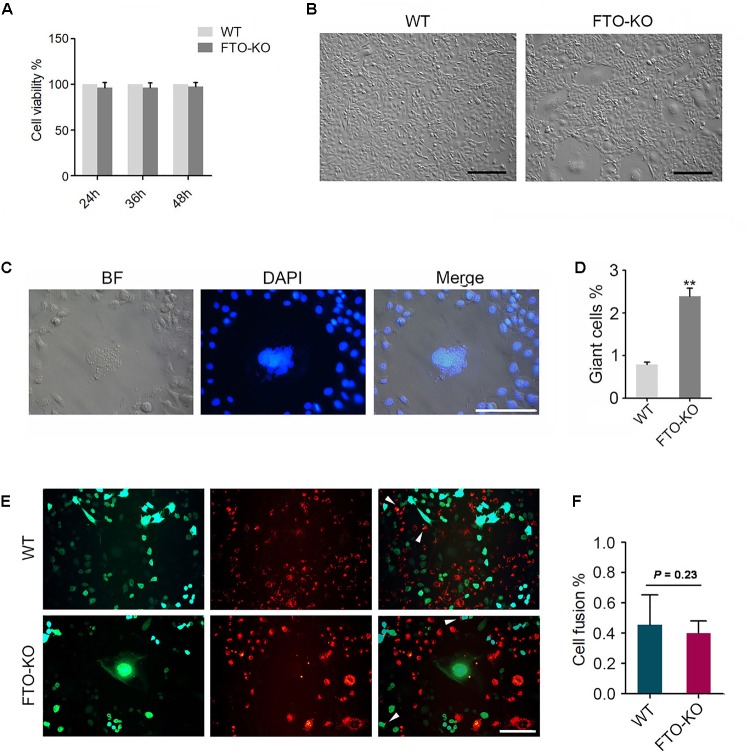
Phenotypes of FTO depletion in spermatogonia. **(A)** Effect of FTO depletion on cell viability. **(B)** Image of morphology of wild-type cells and FTO-KO cells. Bar = 100 μM. **(C)** Morphology of nuclei of giant cells and normal cells. **(D)** Percentage of giant cells were shown in column diagram. Data were represented by the mean ± SEM, *n* = 3, ^∗∗^*p* < 0.01. **(E)** Wild-type cells and FTO-KO cells were stained with red and green dye, respectively, and were co-cultured for 48 h. Representative image of cell fusion status. White arrows indicate the fused cells. Scale bar = 20 μm. **(F)** Percentage of cell fusion were shown in column diagram. Data were represented by the mean ± SEM, *n* = 3.

CCK-8 assay was used to detect cell viability. FTO depletion did not affect cell viability (Figure 2A). The morphology in the FTO-KO cells was markedly different from the Cas9-transfected controls, which was characterized by an increasing population of cells with large cell sizes and spreading areas (Figure 2B). In general, a small number of giant cells can be found in the wild type cells, which was considered as the binucleated spermatocytes. Un-expectedly, in the FTO-KO cells the rate and size of giant cells dramatically increased (Figure 2D). To further investigate the giant cells in detail, we stained cell nuclei using DAPI. As shown in Figure 2C, giant cells were aneuploidies that contained large and irregular nuclei. Flow cytometry analysis showed that the ratio of aneuploidies was significantly increased in FTO-KO cells compared with WT cells (Supplementary Figure S1). These results suggested that FTO deletion caused aneuploidy formation in spermatogonia.

### FTO Depletion Suppresses Chromosome Segregation

Either cell fusion or abnormal chromosome segregation probably leads to aneuploidy formation. To determine whether the increase in aneuploidy proportion was caused by cell fusion, we stained the cells using the double fluorescent tracer assay. The cells stained with red dye and the one stained with green dye were mixed and cultured for 48 h, the fused cells showed bifluorescence (Figure 2E). However, the proportion of fused cells in FTO-KO cells was not different with that in wild-type cells (Figure 2F), indicating that the giant cells were not induced by cell fusion.

**FIGURE 3:**
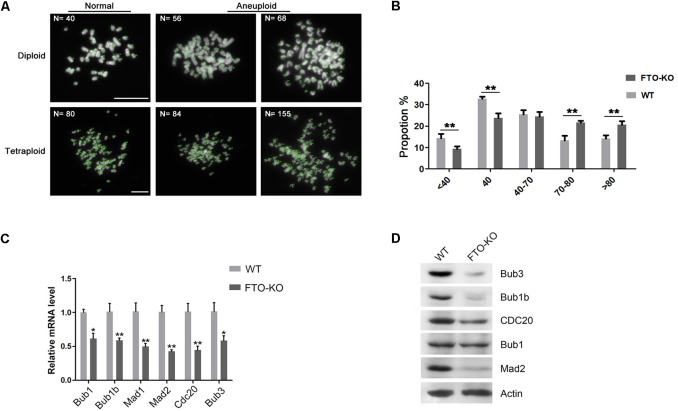
Effect of FTO depletion on chromosome segregation. **(A)** Representative images of normal diploidy and aneuploidy chromosome spread in FTO-KO cells. **(B)** Chromosome numbers from individual metaphase spreads of FTO-KO and wild-type cells. **(C)** Relative mRNA expression of MCC components. Data were represented by the mean ± SEM, *n* = 3, ^∗^*p* < 0.05, ^∗∗^*p* < 0.01. **(D)** Western blot analysis of MCC components expression in wild-type cells and FTO-KO cells. β-actin was used as the loading control.

To investigate whether the aneuploidy was induced by abnormal chromosome segregation, we counted the chromosomes in wild-type cells and FTO-KO cells through the chromosome spreading assay. Interestingly, chromosome number in FTO-KO cells showed a significant increase, compared with wild-type cells (Figures 3A,B). The mitotic checkpoint complex (MCC) is the effector of the spindle assembly checkpoint (SAC) that prevents cells from undergoing cytokinesis when the spindle is assembled improperly with chromosome at metaphase (Lara-Gonzalez et al., 2012). Previous studies have reported that dys-regulation of MCC components resulted in chromosomal instability and aneuploidy (Kapanidou et al., 2015). Here, we hypothesized that FTO depletion induced the formation of aneuploidy due to aberrant expression of MCC. To verify it, we detected the expression of the core MCC components Mad1, Mad2, Bub1, Bub1b, Bub3, and Cdc20. Interestingly, the expression of all detected MCC components significantly decreased in FTO-KO cells both in mRNA and protein levels (Figures 3C,D). These results suggested that FTO deletion suppressed chromosome segregation and induced aneuploidy formation through up-regulation of MCC expression.

### FTO Depletion Arrests G2/M Transition

**FIGURE 4:**
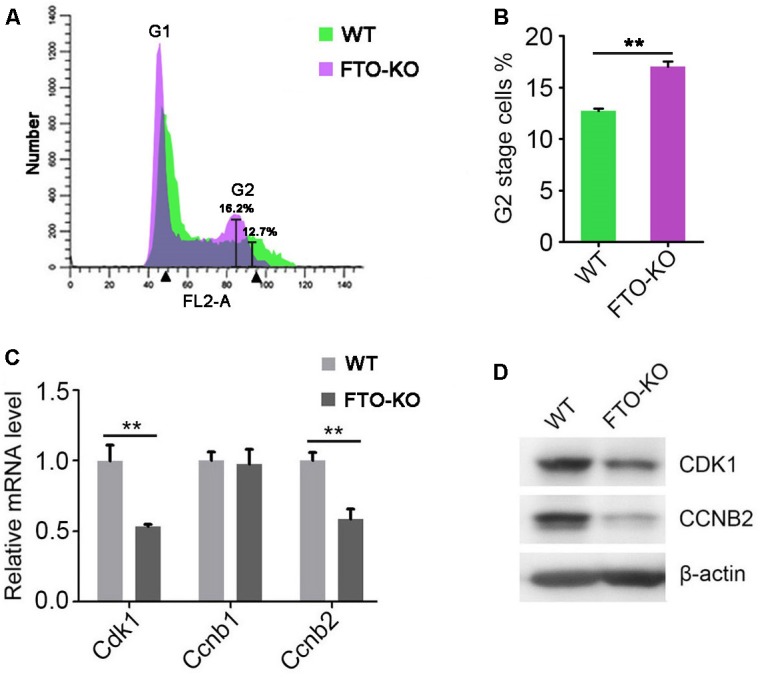
Effect of FTO depletion on cell cycle. **(A)** Cell cycle of FTO-KO and wild-type cells were analyzed by flow cytometry. **(B)** Percentage of G2 stage cells were shown in column diagram. Data were represented by the mean ± SEM, *n* = 3, ^∗∗^*p* < 0.01. **(C)** Relative mRNA level of genes involved in G2/M transition. Data were represented by the mean ± SEM, *n* = 3, ^∗∗^*p* < 0.01. **(D)** Western blot analysis of proteins involved in G2/M transition. β-actin was used as the loading control.

Previous studies have shown that m^6^A methylation is correlated with cell cycle progress during oocyte meiotic maturation (Qi et al., 2016). Therefore, we presumed that FTO regulates cell cycle in spermatogonia. To this end, we analyzed the cell cycle by flow cytometry. Interestingly, we found that the proportion of G2 stage cells significantly increased in FTO-KO cells, compared with wild-type cells (Figures 4A,B). We next detected the expression of core regulatory proteins involved in G2/M transition. As shown in Figures 4C,D, the expression of CDK1 and CCNB2 was significantly down-regulated in FTO-KO cells, indicating that FTO modulated G2/M transition through regulating the expression of Cdk1/Ccnb2 complex.

### FTO Regulates Cell Cycle and Aneuploidy Formation Through the m^6^A Demethylase Activity

**FIGURE 5:**
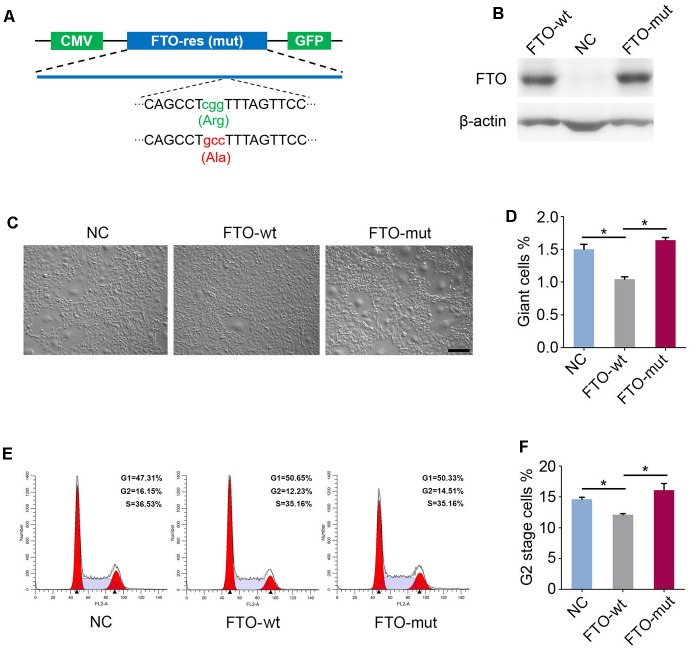
Fat mass and obesity-associated protein regulates giant cell formation and G2/M transition dependent on the m^6^A demethylase activity. **(A)** Schematic diagram of FTO-wt and FTO-mut plasmids. Green and red letters represent the mutation of 313R to A. **(B)** Western blot analysis of FTO expression in the FTO-wt cells and the FTO-mut cells. β-actin was used as the loading control. **(C)** Morphology of FTO-wt cells and FTO-mut cells. Bar = 100 μm. **(D)** Percentage of giant cells were shown in column diagram. Data were represented by the mean ± SEM, *n* = 3, ^∗∗^*p* < 0.01. **(E)** Cell cycle of FTO-wt and FTO-mut cells were analyzed by flow cytometry. **(F)** Percentage of G2 stage cells were shown in column diagram. Data were represented by the mean ± SEM, *n* = 3, ^∗^*p* < 0.05.

Previous studies have reported that mutation of the critical amino acid residue 313R to A (R313A) in the catalytic center of FTO protein can completely ablate its m^6^A demethylase activity (Zhao et al., 2014). Hence, to investigate whether the FTO knockout phenotype in spermatogonia is due to its m^6^A demethylase activity, we constructed two lentivirus vectors that expressed wild-type FTO (named FTO-wt) and R313A mutant FTO (named FTO-mut), respectively (Figure 5A). We next established three cell lines by transfection of the FTO-wt, the FTO-mut and the control (GFP) lentivirus to the FTO-KO cells, respectively. Western blot analysis showed that the FTO expression was rescued in FTO-wt and FTO-mut cells, but not control cells (Figure 5B). We next detected the proportion of aneuploidy and G2 stage cells in the three cell lines. Interestingly, the rate of aneuploidy and G2 stage cells in FTO-wt group was significantly less than those in the FTO-mut and control cells, indicating that the FTO depletion phenotype could be partially rescued by wild-type FTO but not mutant FTO (Figures 5C–F). These results suggested that FTO regulated cell cycle in spermatogonia mainly through its m^6^A demethylase activity.

**FIGURE 6:**
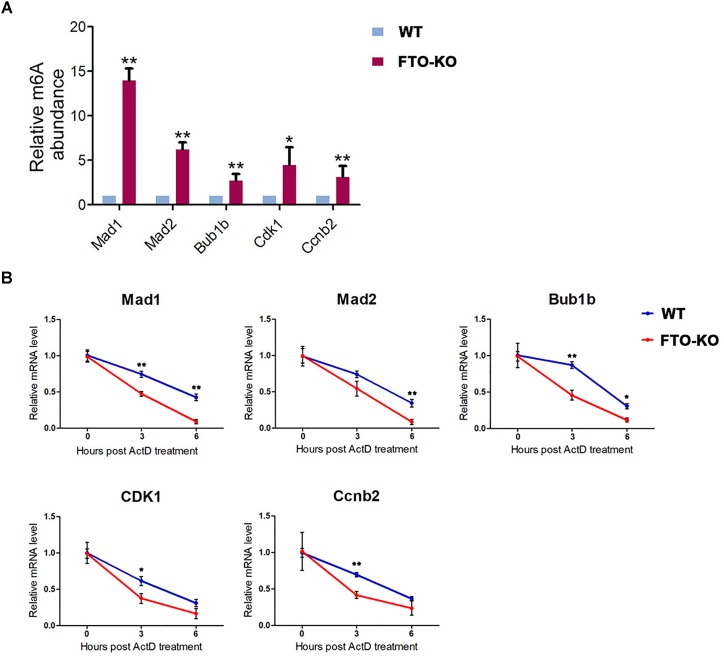
Fat mass and obesity-associated protein targets the m^6^A modification on the key components of MCC and G2/M regulators. **(A)** mRNA isolated from wild-type cells and FTO-KO cells were subjected to m^6^A-IP. The m^6^A positive mRNA were detected via quantitative PCR. Y axes represent the relative mRNA level in IP-mRNA compared to input. Data were represented by the mean ± SEM, *n* = 3, ^∗^*p* < 0.05, ^∗∗^*p* < 0.01. **(B)** Cells were treated with 5 μg/mL actinomycin and harvested for RNA extraction. Target mRNA level were quantitated by q-PCR. X axes represent hours post treatment. Y axes represent the remained mRNA level. Data were represented by the mean ± SEM, *n* = 3, ^∗^*p* < 0.05, ^∗∗^*p* < 0.01.

To verify whether FTO deletion leads to increase of m^6^A level in the transcripts of target genes, we performed m^6^A-IP-qPCR. As shown in Figure 6A, in the m^6^A-IP transcripts, the abundance of Bub1b, Mad1, Mad2, Cdk1 and Ccnb2 was significantly up-regulated in FTO depletion group, while Bub1, Cdc20, and Bub3 were undetectable under the sensitivity of q-PCR, suggesting that m^6^A level in the transcripts of Bub1b, Mad1, Mad2, Cdk1, and Ccnb2 is elevated due to FTO knockout.

To further demonstrate whether the increased m^6^A level accelerates the degradation of target mRNAs, we performed an RNA decay assay. Cells were treated with 5 μg/mL actinomycin D for 0, 3, and 6 h and harvested for RNA extraction. The remained mRNA level was normalized by real-time quantitative PCR. As shown in Figure 6B, mRNA stability of Mad1, Mad2, Bub1b, CDK1, and Ccnb2 were significantly decreased after FTO depletion. These results suggested that FTO regulated the expression of target transcripts through the regulation of RNA stability.

Together, these data indicate that FTO directly regulates the expression of the core MCC components and G2/M regulators through the m^6^A/RNA decay pathway, thus regulating cell cycle and mitosis checkpoint in spermatogonia.

## Discussion

Spermatogenesis is a highly dynamic developmental process involving intricate regulation of gene expression. The significance of m^6^A in spermatogenesis has increasingly been unraveled. FTO, the first discovered m^6^A demethylase, regulates RNA splicing, stability or translation, thereby making a difference in cell fate determination (Li L. et al., 2017). However, FTO function in spermatogonia remain unclear. Here, we established a Fto-null mouse spermatogonial cell line using CRISPR/Cas9 system. We found that FTO deletion led to aneuploidy formation and G2/M arrest. We further demonstrated that FTO demethylated five transcripts of core MCC components and G2/M regulators. The findings suggest that FTO regulates chromosome segregation and cell cycle progression via m^6^A demethylase activity.

Accurate segregation of duplicated chromosomes is indispensable for the cell division. The accurate chromosome segregation relies on precise temporal regulation of sequential processes including the orientation of bipolar spindle, the attachment of kinetochore and microtubules and the separation of daughter cells during cytokinesis (Thompson et al., 2010). Error occurred at any step may lead to chromosome mis-segregation and aneuploidy formation (Meraldi, 2016). The mitotic checkpoint is a safeguard mechanism against the aneuploidy formation (London and Biggins, 2014). When chromosomes fails to assemble with spindle, the checkpoint activates to inhibit the downstream anaphase promoting complex (APC/C), resulting in prevention of cells from entry the next cell cycle (Thompson et al., 2010). The importance of MCC in the regulation of chromosome instability have been largely reported (Lara-Gonzalez et al., 2012). Chromosome segregation errors in mitosis are the most common cause for aneuploidies formation *in vitro*, as well as in clinical cancer samples (van Jaarsveld and Kops, 2016). Hence, the MCC components have been selected as promising targets for the therapy of cancers (Tanaka and Hirota, 2016). In the present study, we found that FTO regulates the expression of core MCC components, thus regulating chromosome segregation. The roles of FTO and m^6^A in the regulation of chromosome stability have not been reported yet. Therefore, our results suggested that FTO may play important roles in the progression of seminoma for the first time. It will be interesting to deeply investigate the functions of FTO in seminoma carcinogenesis.

The Cdk1/Ccnb complex is the main composition of the maturation-promoting factor (MPF) that triggers the G2/M transition (Adhikari and Liu, 2014). The role of Cdk1/Ccnb in the regulation of metaphase arrest during oogenesis have been well documented (Turner, 2015). In contrast, studies on the function of MFP in spermatogenesis are limited. Clement et al. (2015) reported that decreased expression of Cdk1 caused late meiotic arrest and infertility in mice. Recent studies showed that FTO regulates the expression of CDK2 and CCNB2, thus affecting cell cycle progression during adipogenesis (Wu et al., 2018). In the present study, we showed that FTO depletion led to decreased expression of the Cdk1 and Ccnb2, resulting in G2/M arrest in spermatogonia.

A few studies have reported the significance of m^6^A relative proteins in spermatogenesis (Zheng et al., 2013; Hsu et al., 2017; Lin et al., 2017). The underlying mechanisms of how m^6^A regulates the spermatogenesis remain obscure. Previous studies mainly analyzed the global m^6^A methylome, combined the variation of m^6^A peaks with the differentiation in global expression or splicing of transcriptome, thus determined the function of m^6^A in stability or splicing of target transcripts (Xu et al., 2017). In the present study, we detected the m^6^A level of target genes via the m^6^A-IP-qPCR assay, which can precisely reveal the m^6^A variation on target transcripts. We found that three transcripts of the core MCC components (Bub1b, Mad1, and Mad2) and two transcripts of the G2/M regulatory proteins (Cdk1 and Ccnb2), were directly targeted by FTO. We also demonstrated that the increased m^6^A level retarded the stability of target transcripts. Recent reports have shown that FTO simultaneously demethylates m^6^A and m^6^A_m_ in mammalian cells. To further elucidate the mechanism by which FTO knockout leads to the phenotypes, it will be interesting to detect m^6^A_m_ through the miCLIP-seq (Mauer et al., 2017; Wei et al., 2018). Additionally, our results showed that m^6^A modification did not occur in the transcripts of Bub1, Bub3, and Cdc20, indicating that FTO regulates the three genes through other pathways. It is no doubt that changes on gene expression can be global after FTO depletion. Though the target genes we focused on are directly associated with the phenotypes, contributions of other differentially expressed genes through other pathways should not be ignored. The mechanisms of m^6^A on the regulation of gene expression are comprehensive. Changes in m^6^A level can make differences in mRNA decay, splicing or translation, which relies on the recognition of different readers. Here we mainly focused on the effects of FTO on the decay of target transcripts. Hence, to gain deeper insights into the regulatory role of FTO to the phenotype, RNA-seq combined with splicing analysis and translation efficiency assay will be necessary.

Male infertility has been becoming a worldwide problem in recent years (Mascarenhas et al., 2012). To understand the underlying mechanisms of spermatogenesis is important for the precise therapy of male infertility. As spermatogonia are the precursor of male germ cells, to elucidate the regulation of spermatogonia homeostasis is important for understanding male infertility. The present study first revealed the role of RNA demethylase FTO in the regulation of chromosome instability and cell cycle progression in spermatogonia, thus giving novel insights into the role of RNA methylation in spermatogenesis and potentially, in seminoma progression. Our studies are limited to the functions of FTO in immortalized cell line.It will be important to generate conditional knockout mice to gain better understandings of the roles of FTO plays in spermatogenesis.

## Conclusion

In conclusion, knockout of FTO triggered aberrant chromosome segregation and cell cycle arrest, which could be partially rescued by wild-type FTO but not mutant FTO. FTO depletion elevated the m^6^A level of core MCC components and G2/M regulators. Therefore, FTO regulates cell cycle and mitosis checkpoint in spermatogonia through the m^6^A/mRNA degradation pathway. Our findings give novel insights into the role of RNA methylation in spermatogenesis.

## Data Availability Statement

All datasets (generated/analyzed) for this study are included in the manuscript.

## Author Contributions

TH and WZ conceived and designed the experiments. TH, QG, TF, and JG performed the experiments. TH analyzed the data. TH, WZ, and YZ wrote the manuscript.

## Conflict of Interest Statement

The authors declare that the research was conducted in the absence of any commercial or financial relationships that could be construed as a potential conflict of interest.

## References

[B1] Adhikari D. Liu K. (2014). The regulation of maturation promoting factor during prophase I arrest and meiotic entry in mammalian oocytes. *Mol. Cell. Endocrinol.* 382 480–487. 10.1016/j.mce.2013.07.027 23916417

[B2] Alarcon C. R. Lee H. Goodarzi H. Halberg N. Tavazoie S. F. (2015). N6-methyladenosine marks primary microRNAs for processing. *Nature* 519 482–485. 10.1038/nature14281 25799998 PMC4475635

[B3] Batista P. J. Molinie B. Wang J. Qu K. Zhang J. Li L. (2014). m(6)A RNA modification controls cell fate transition in mammalian embryonic stem cells. *Cell Stem Cell* 15 707–719. 10.1016/j.stem.2014.09.019 25456834 PMC4278749

[B4] Clement T. M. Inselman A. L. Goulding E. H. Willis W. D. Eddy E. M. (2015). Disrupting cyclin dependent kinase 1 in spermatocytes causes late meiotic arrest and infertility in mice. *Biol. Reprod.* 93:137. 10.1095/biolreprod.115.134940 26490841 PMC4712696

[B5] Dominissini D. Moshitch-Moshkovitz S. Schwartz S. Salmon-Divon M. Ungar L. Osenberg S. (2012). Topology of the human and mouse m6A RNA methylomes revealed by m6A-seq. *Nature* 485 201–206. 10.1038/nature11112 22575960

[B6] Du K. Zhang L. Lee T. Sun T. (2018). m(6)A RNA methylation controls neural development and is involved in human diseases. *Mol. Neurobiol.* 10.1007/s12035-018-1138-1 [Epub ahead of print]. 29909453

[B7] Engel M. Eggert C. Kaplick P. M. Eder M. Roh S. Tietze L. (2018). The role of m(6)A/m-RNA methylation in stress response regulation. *Neuron* 99:e389. 10.1016/j.neuron.2018.07.009 30048615 PMC6069762

[B8] Fischer J. Koch L. Emmerling C. Vierkotten J. Peters T. Bruning J. C. (2009). Inactivation of the Fto gene protects from obesity. *Nature* 458 894–898. 10.1038/nature07848 19234441

[B9] Fok K. L. Chen H. Ruan Y. C. Chan H. C. (2014). Novel regulators of spermatogenesis. *Semin. Cell Dev. Biol.* 29 31–42. 10.1016/j.semcdb.2014.02.008 24594193

[B10] Hamra F. K. Schultz N. Chapman K. M. Grellhesl D. M. Cronkhite J. T. Hammer R. E. (2004). Defining the spermatogonial stem cell. *Dev. Biol.* 269 393–410. 10.1016/j.ydbio.2004.01.027 15110708

[B11] Hsu P. J. Zhu Y. F. Ma H. H. Guo Y. H. Shi X. D. Liu Y. Y. (2017). Ythdc2 is an N-6-methyladenosine binding protein that regulates mammalian spermatogenesis. *Cell Res.* 27 1115–1127. 10.1038/cr.2017.99 28809393 PMC5587856

[B12] Kanatsu-Shinohara M. Shinohara T. (2013). Spermatogonial stem cell self-renewal and development. *Annu. Rev. Cell. Dev. Biol.* 29 163–187. 10.1146/annurev-cellbio-101512-122353 24099084

[B13] Kapanidou M. Lee S. Bolanos-Garcia V. M. (2015). BubR1 kinase: protection against aneuploidy and premature aging. *Trends Mol. Med.* 21 364–372. 10.1016/j.molmed.2015.04.003 25964054

[B14] Landfors M. Nakken S. Fusser M. Dahl J. A. Klungland A. Fedorcsak P. (2016). Sequencing of FTO and ALKBH5 in men undergoing infertility work-up identifies an infertility-associated variant and two missense mutations. *Fertil. Steril.* 105:e1175. 10.1016/j.fertnstert.2016.01.002 26820768

[B15] Lara-Gonzalez P. Westhorpe F. G. Taylor S. S. (2012). The spindle assembly checkpoint. *Curr. Biol.* 22 R966–R980. 10.1016/j.cub.2012.10.006 23174302

[B16] Li H. B. Tong J. Zhu S. Batista P. J. Duffy E. E. Zhao J. (2017). m(6)A mRNA methylation controls T cell homeostasis by targeting the IL-7/STAT5/SOCS pathways. *Nature* 548 338–342. 10.1038/nature23450 28792938 PMC5729908

[B17] Li L. Zang L. Zhang F. Chen J. Shen H. Shu L. (2017). Fat mass and obesity-associated (FTO) protein regulates adult neurogenesis. *Hum. Mol. Genet.* 26 2398–2411. 10.1093/hmg/ddx128 28398475 PMC6192412

[B18] Li Z. J. Weng H. Y. Su R. Weng X. C. Zuo Z. X. Li C. Y. (2017). FTO plays an oncogenic role in acute myeloid leukemia as a N-6-Methyladenosine RNA demethylase. *Cancer Cell* 31 127–141. 10.1016/j.ccell.2016.11.017 28017614 PMC5234852

[B19] Lin Z. Hsu P. J. Xing X. Fang J. Lu Z. Zou Q. (2017). Mettl3-/Mettl14-mediated mRNA N(6)-methyladenosine modulates murine spermatogenesis. *Cell Res.* 27 1216–1230. 10.1038/cr.2017.117 28914256 PMC5630681

[B20] Liu J. Z. Yue Y. N. Han D. L. Wang X. Fu Y. Zhang L. (2014). A METTL3-METTL14 complex mediates mammalian nuclear RNA N-6-adenosine methylation. *Nat. Chem. Biol.* 10 93–95. 10.1038/nchembio.1432 24316715 PMC3911877

[B21] London N. Biggins S. (2014). Signalling dynamics in the spindle checkpoint response. *Nat. Rev. Mol. Cell Biol.* 15 735–747. 10.1038/nrm3888 25303117 PMC4283840

[B22] Mascarenhas M. N. Flaxman S. R. Boerma T. Vanderpoel S. Stevens G. A. (2012). National, regional, and global trends in infertility prevalence since 1990: a systematic analysis of 277 health surveys. *PLoS Med.* 9:e1001356. 10.1371/journal.pmed.1001356 23271957 PMC3525527

[B23] Mauer J. Luo X. Blanjoie A. Jiao X. Grozhik A. V. Patil D. P. (2017). Reversible methylation of m(6)Am in the 5′ cap controls mRNA stability. *Nature* 541 371–375. 10.1038/nature21022 28002401 PMC5513158

[B24] Meraldi P. (2016). Centrosomes in spindle organization and chromosome segregation: a mechanistic view. *Chromosom. Res.* 24 19–34. 10.1007/s10577-015-9508-2 26643311

[B25] Niu Y. Zhao X. Wu Y. S. Li M. M. Wang X. J. Yang Y. G. (2013). N6-methyl-adenosine (m6A) in RNA: an old modification with a novel epigenetic function. *Genomics Proteomics Bioinformatics* 11 8–17. 10.1016/j.gpb.2012.12.002 23453015 PMC4357660

[B26] Qi S. T. Ma J. Y. Wang Z. B. Guo L. Hou Y. Sun Q. Y. (2016). N6-Methyladenosine sequencing highlights the involvement of mRNA methylation in oocyte meiotic maturation and embryo development by regulating translation in Xenopus laevis. *J. Biol. Chem.* 291 23020–23026. 10.1074/jbc.M116.748889 27613873 PMC5087722

[B27] Tanaka K. Hirota T. (2016). Chromosomal instability: a common feature and a therapeutic target of cancer. *Biochim. Biophys. Acta Rev. Cancer* 1866 64–75. 10.1016/j.bbcan.2016.06.002 27345585

[B28] Thompson S. L. Bakhoum S. F. Compton D. A. (2010). Mechanisms of chromosomal instability. *Curr. Biol.* 20 R285–R295. 10.1016/j.cub.2010.01.034 20334839 PMC3781365

[B29] Turner J. M. (2015). Meiotic silencing in mammals. *Annu. Rev. Genet.* 49 395–412. 10.1146/annurev-genet-112414-055145 26631513

[B30] van Jaarsveld R. H. Kops G. J. P. L. (2016). Difference Makers: chromosomal Instability versus Aneuploidy in Cancer. *Trends Cancer* 2 561–571. 10.1016/j.trecan.2016.09.003 28741487

[B31] Wang X. Lu Z. Gomez A. Hon G. C. Yue Y. Han D. (2014). N6-methyladenosine-dependent regulation of messenger RNA stability. *Nature* 505 117–120. 10.1038/nature12730 24284625 PMC3877715

[B32] Wang X. Zhao B. S. Roundtree I. A. Lu Z. Han D. Ma H. (2015). N(6)-methyladenosine modulates messenger RNA translation efficiency. *Cell* 161 1388–1399. 10.1016/j.cell.2015.05.014 26046440 PMC4825696

[B33] Wang Y. Li Y. Yue M. H. Wang J. Kumar S. Wechsler-Reya R. J. (2018). N-6-methyladenosine RNA modification regulates embryonic neural stem cell self-renewal through histone modifications. *Nat. Neurosci.* 21 1139–1139. 10.1038/s41593-018-0169-2 29880878

[B34] Wei J. Liu F. Lu Z. Fei Q. Ai Y. He P. C. (2018). Differential m(6)A, m(6)Am, and m(1)A demethylation mediated by FTO in the cell nucleus and cytoplasm. *Mol. Cell.* 71:e975. 10.1016/j.molcel.2018.08.011 30197295 PMC6151148

[B35] Wu R. Liu Y. Yao Y. Zhao Y. Bi Z. Jiang Q. (2018). FTO regulates adipogenesis by controlling cell cycle progression via m(6)A-YTHDF2 dependent mechanism. *Biochim. Biophys. Acta. Mol. Cell. Biol. Lipids* 1863 1323–1330. 10.1016/j.bbalip.2018.08.008 30305247

[B36] Xiang Y. Laurent B. Hsu C. H. Nachtergaele S. Lu Z. Sheng W. (2017). RNA m(6)A methylation regulates the ultraviolet-induced DNA damage response. *Nature* 543 573–576. 10.1038/nature21671 28297716 PMC5490984

[B37] Xu K. Yang Y. Feng G. H. Sun B. F. Chen J. Q. Li Y. F. (2017). Mettl3-mediated m(6)A regulates spermatogonial differentiation and meiosis initiation. *Cell Res.* 27 1100–1114. 10.1038/cr.2017.100 28809392 PMC5587845

[B38] Zhao X. Yang Y. Sun B. F. Shi Y. Yang X. Xiao W. (2014). FTO-dependent demethylation of N6-methyladenosine regulates mRNA splicing and is required for adipogenesis. *Cell Res.* 24 1403–1419. 10.1038/cr.2014.151 25412662 PMC4260349

[B39] Zheng G. Q. Dahl J. A. Niu Y. M. Fedorcsak P. Huang C. M. Li C. J. (2013). ALKBH5 is a mammalian RNA demethylase that impacts RNA metabolism and mouse fertility. *Mol. Cell* 49 18–29. 10.1016/j.molcel.2012.10.015 23177736 PMC3646334

[B40] Zhu T. Roundtree I. A. Wang P. Wang X. Wang L. Sun C. (2014). Crystal structure of the YTH domain of YTHDF2 reveals mechanism for recognition of N6-methyladenosine. *Cell Res.* 24 1493–1496. 10.1038/cr.2014.152 25412661 PMC4260350

