# Supplementary material for: FTO Knockout Causes Chromosome Instability and G2/M Arrest in Mouse GC-1 Cells
Source: Front Genet. 2019 Jan 21;9:732. doi: 10.3389/fgene.2018.00732 (PMC6348250; doi:10.3389/fgene.2018.00732)
Supplement: Supplementary file 1 [file Data_Sheet_1.PDF]

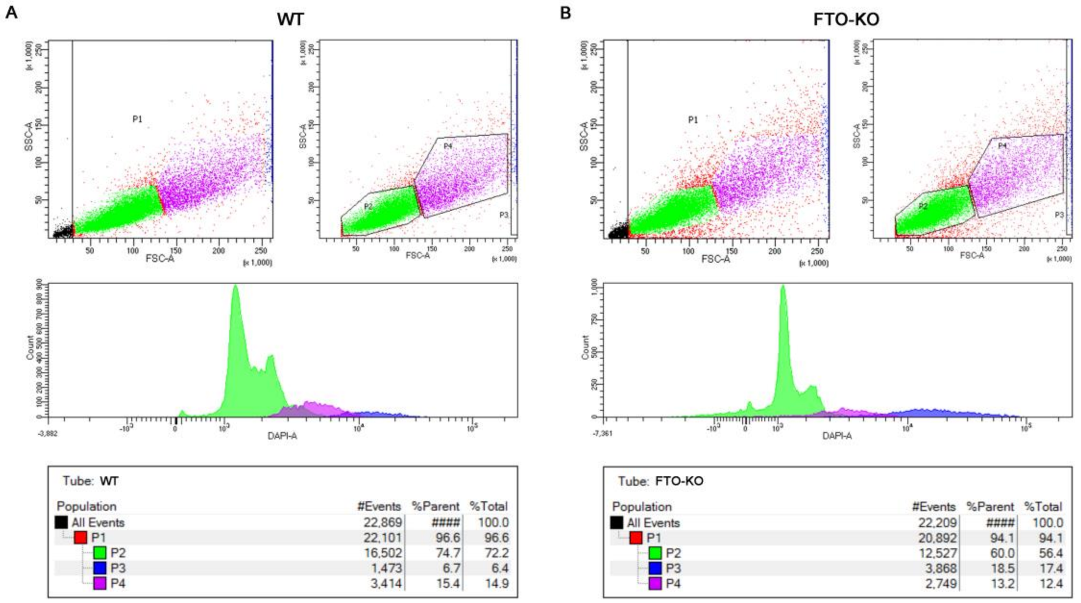

Figure S1. Flow cytometry analysis of aneuploidy. Cell nucleus were stained with DAPI and cells were sorted by the fluorescence strength. P1 represent the population of total available cells. P2 represent the population of normal diploid and tetraploid cells. P3 and P4 represent the population of aneuploidy cells (P3 positive cells are located out of the indication range, which represent the multinuclear cells). **A.** Analysis of WT cells. **B.** Analysis of FTO-KO cells. Data show that the proportion of P3 positive cells is significantly increased in FTO-KO cells compared with WT.
