# Supplementary material for: FTO Knockout Causes Chromosome Instability and G2/M Arrest in Mouse GC-1 Cells
Source: Front Genet. 2019 Jan 21;9:732. doi: 10.3389/fgene.2018.00732 (PMC6348250; doi:10.3389/fgene.2018.00732)
Supplement: Supplementary file 2 [file Table_1.pdf]

Supplymentary materials

Table S1. Oligo information

Oligo name	Sequences (5’ - 3’)
Mad1-q-F	AGCCGTCTGGAACAGGAAAAG
Mad1-q-R	GGGTTAAGGCTCATGTGTAGCA
Mad2-q-F	GTGGCCGAGTTTTTCTCATTTG
Mad2-q-R	AGGTGAGTCCATATTTCTGCACT
Bub1-q-F	AGAATGCTCTGTCAGCTCATCT
Bub1-q-R	TGTCTTCACTAACCCACTGCT
Cdc20-q-F	TTCGTGTTCGAGAGCGATTTG
Cdc20-q-R	ACCTTGGAACTAGATTTGCCAG
FTOres-F	GAATCTAGAATGAAGCGCGTCCAGAC
FTOres-R	GGAGAATTCTGCTGGAAGCAAGATCCTAG
ftoR313A-F	GCTGGCTCACAGCCTGCGTTTAGTTCCACTCACCG
ftoR313A-R	GCGTGAGTGGAACTAAACGCAGGCTGTGAGCCAGC
BUB3-q-F	GATGGCATCTCCTCGGTTAAG
BUB3-q-R	AATTGGCGGGCACATCGTAG
CCNB1-q-F	CTTGCAGTGAGTGACGTAGAC
CCNB1-q-R	CCAGTTGTCGGAGATAAGCATAG
CCNB2-q-F	GCCAAGAGCCATGTGACTATC
CCNB2-q-R	CAGAGCTGGTACTTTGGTGTTC
